# Improved RSV Neutralization Assay Using Recombinant RSV Expressing Reporter Fluorescent Protein

**DOI:** 10.3390/mps8030060

**Published:** 2025-06-04

**Authors:** Yutaro Yamagata, Michiko Toizumi, Jean-Francois Eleouet, Marie-Anne Rameix-Welti, Makoto Takeda, Lay-Myint Yoshida

**Affiliations:** 1 Department of Pediatric Infectious Diseases, Institute of Tropical Medicine, Nagasaki University, Nagasaki 852-8523, Japan; y_yutaro@nagasaki-u.ac.jp (Y.Y.); toizumi@nagasaki-u.ac.jp (M.T.); 2 School of Tropical Medicine and Global Health, Nagasaki University, Nagasaki 852-8523, Japan; 3 Unité de Virologie et Immunologie Moléculaires (VIM), Université Paris-Saclay, INRAE, 78350 Jouy-en-Josas, France; jean-francois.eleouet@inrae.fr; 4 Molecular Mechanisms of Multiplication of Pneumoviruses Unit (M3P), Institut Pasteur, Université Paris-Saclay, Université de Versailles St. Quentin, Université Paris Cité, UMR 1173 (2I), INSERM, Centre National de Reference Virus des Infections Respiratoires (CNR VIR), Assistance Publique des Hôpitaux de Paris, Paris, France; marie-anne.rameix-welti@pasteur.fr; 5 Faculty of Medicine, Graduate School of Medicine, The University of Tokyo, Tokyo 113-0033, Japan; mtakeda@m.u-tokyo.ac.jp; 6 Graduate School of Biomedical Sciences, Nagasaki University, Nagasaki 852-8523, Japan

**Keywords:** RSV, recombinant virus, fluorescent protein, neutralization assay

## Abstract

Human respiratory syncytial virus (RSV) causes acute respiratory illness, attributing to deaths among young children and older adults worldwide. RSV neutralization assay is an important tool to measure RSV neutralization antibody that can prevent infection and severe complication of RSV. Conventional RSV neutralization assays have some limitations of speed and cost, especially for expensive kits, reagents or instruments required for detection. To solve this problem, this paper describes an improved simple and economical RSV neutralization assay protocol using recombinant RSV (rRSV) expressing reporter fluorescent protein to measure RSV growth as reporter activity with plate reader. The condition of 3 days culture demonstrated sufficient fluorescent activity even when small amounts of rRSV were used to inoculate Hep-2 cells. In addition, white 96-well cell culture plate showed better stable reporter activities than black plate. Furthermore, RSV neutralization assay protocol using rRSV-reporter fluorescent protein demonstrated similar signal detection capacity for RSV antibody titer detection compared to other protocols, such as rRSV-Luciferase and ELISA assay. The new RSV neutralization assay protocol can be applied to RSV antibody titration of numerous samples necessary for RSV surveillance or antiviral testing.

## 1. Introduction

Human respiratory syncytial virus (RSV) is one-segment negative-strand RNA virus, which belongs to Pneumoviridae. RSV is one of the leading causes of acute respiratory illness in young children, immunocompromised patients, and older adults worldwide. RSV-attributing deaths of 0–5 years aged children were estimated to be over 100,000 globally in 2019, more than 97% of which occurred in low-income and middle-income countries [1]. RSV incidence rates and hospitalization rates among older adults in industrialized countries were estimated to be 600.7 cases per 100,000 person-years and 157 hospitalizations per 100,000 person-years, respectively [2]. RSV often causes reinfection because recovered RSV-patients lose RSV-neutralization antibody gradually [2,3] and RSV-antigenicity is changed by mutations of RSV fusion protein (F) and glycoprotein (G) [4]. Although some anti-RSV vaccines and drugs are developed [5], they still have challenges for world-wide usage. For example, recombinant RSV F-protein vaccine Arexvy (GSK, London, UK) is available for use in people over 60 years old [6], while recombinant RSV pre-F vaccine Abrysvo (Pfizer, Brooklyn, NY, USA) can be used for pregnant women [7] and people over 60 years old [8]. Monoclonal antibodies to prevent severe RSV infection such as Palivizumab (Medimmune, Gaithersburg, MD, USA) and Nirsevimab (AstraZeneca, Cambridge, UK) are available to be used in infants and young children especially with high risk. However, due to the high price and availability, their usage is limited, especially in low and middle-income countries. Further research for ARI caused by RSV is necessary to improve RSV vaccines and drugs development.

RSV neutralization assay is one of the important tools to measure RSV antiviral antibody titer in patients’ blood serum samples, which is helpful for examination of vaccine effectiveness. Enzyme Immunoassay (EIA) protocol-based assays that use an antigen-coated plates to detect antibodies in samples, are simpler and less expensive but they do not measure the neutralization antibody titer [9]. In conventional neutralization assays, serum samples are diluted serially with medium, mixed with RSV and incubated for neutralizing reaction. A mixture of diluted sera with RSV was introduced into the cell culture. After several days culture of infected cells, 50% inhibitory concentration (IC50), antiserum sample concentration of the sample reducing viral growth by half of no antiserum sample, is usually calculated as IC50 antibody titer. There are some protocols available to measure viral growth of sample-virus mixture, for example, plaque counting [10,11,12], quantitative PCR, qPCR [13] or Enzyme-Linked Immunosorbent Assay (ELISA) [14]. Although micro-scale assay is beneficial to save labor when analyzing a larger number of samples like clinical surveillance, plaque counting on micro-well plates like 96-well plates requires a special far expensive ELISPOT-reader machine for detection of small plaques, difficult to accurately detected by naked eyes. qPCR and ELISA protocols need sensitive operations and prepared reagents, such as qPCR SYBR Green and ELISA first antibody targeting viral protein. Simple and affordable RSV micro-neutralization assay protocol that can analyze a large number of samples without using expensive instruments will be useful in clinical and epidemiological surveillance studies.

**Figure 1:**
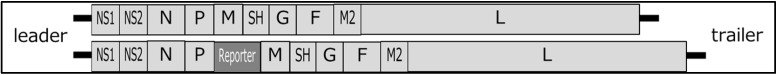
The schematic of RSV (top, about 15.2 kb) and reporter-expressing rRSV (bottom, about >16 kb) genome. Reporter genes such as GFP, mCherry and Luciferase proteins are inserted with gene start signal and gene end signal between RSV P and M genes.

Thus in this study, we used a previously reported recombinant RSV (rRSV) expressing reporter gene [15] for RSV neutralization assay, in which red fluorescent protein mCherry, green fluorescent protein GFP or luciferase protein is inserted as reporter gene between RSV phosphoprotein (P) gene and RSV matrix protein (M) gene (Figure 1). rRSVs show similar viral growth to wild-type RSV and fluorescent or luminescent reporter activity proportional to viral growth in infected cells, which can be measured as fluorescence or luminescence intensity using a plate-reader machine. rRSVs are applied to microbiological study of RSV infectious mechanism [16] and micro-neutralization assay using foci-counting equipment [17] like ELISPOT reader. However, rRSVs are rarely applied to epidemiological study like RSV surveillance, which needs simple and affordable assay protocol. In this study, first, we used three different culture times and two different 96 well plates for fluorescence analysis, and then, we examined 4 rRSVs and different detection systems to set up a high-throughput rRSV neutralization assay that can simultaneously analyze a large set of samples with manual operation and a simple fluorescent plate-reader without foci-counting function.

## 2. Materials and Methods

### 2.1. Cells

Hep-2 human epithelial cells (#CCL-23, ATCC, Manassas, VA, USA) are maintained in Eagle’s Minimum Essential Medium, EMEM (#051-07615, FujifilmWako, Osaka, Japan) supplemented with 10% Fetal Bovine Serum, FBS (#S1530-500, BioWest, Nuaillé, France) and 1% Penicillin-Streptomycin solution ×100, PS (#168-23191, FujifilmWako) at 37 °C in 5% CO_2_ condition.

### 2.2. Viruses

Four rRSVs, rRSV-wild type having no reporter gene (rRSV-WT), rRSV-Luciferase having luciferase reporter gene (rRSV-Luc), rRSV-GFP and rRSV-mCherry (rRSV-Cherry) having fluorescent protein reporter gene, were provided by Prof. Marie-Anne Rameix-Welti in Universite Versailles St-Quentin, Paris Saclay in France [15] through National Institute of Infectious Diseases (NIID), Tokyo, Japan.

rRSVs were cultured in Hep-2 cells according to previous studies [15,18]. 1 × 10^6^ Hep-2 cells/well, seeded at 37 °C in 5% CO_2_ for 1 day using 6-well plate, were infected with rRSV dilution (MOI = 0.01) at 37 °C in 5% CO_2_ for 2 h with every 30 min tilting. After removing rRSV dilution and supplementation of 2%FBS/EMEM-1%PS, infected cells were cultured at 37 °C in 5% CO_2_ for 48 h. After 48 h, the infected cells were scraped from bottom of wells, and supernatants were collected with scraped cells. The mixture was mixed with 10× RSV preservation buffer (0.5 M HEPES, 1 M MgSO_4_, pH 7.3) using 5 s vortex, and centrifuged for 5 min, 200× *g* at 4 °C. 90% supernatant was moved in another tube, and 10% supernatant-cell precipitation repeated freeze-thaw 3 times using −80 °C ethanol/dry ice and 37 °C water-bath to crash cell precipitation. 90% supernatant and 10% supernatant-cell precipitation were mixed, and then the supernatants were divided into tubes to preserve at −80 °C as cultured RSV stocks.

rRSVs were titrated using plaque assay in triplicate. 2.5 × 10^5^ Hep-2 cells/well, seeded at 37 °C in 5% CO_2_ for 1 day using 24-well plate, were infected with rRSV serial-dilution at 37 °C in 5% CO_2_ for 2 h with every 30 min tilting. After removing rRSV dilution, sterile 1.5% carboxyl methyl cellulose (CMC) gel solution was mixed with 2× DMEM/F-12-2%FBS-2%PS pH-neutralized by NaHCO_3_, and the resulted mixture 1× DMEM/F-12-2%FBS-2%PS −0.75% CMC gel solution was overlaid on the infected cells. The infected cells were cultured for 5 days at 37 °C in 5% CO_2_. The cultured cells were fixed with 10-fold diluted formalin for 1 h at room temperature. The formalin and gel solution were removed, and the fixed cells were washed with D-PBS (-) (FujifilmWako#045-29795) 2 times. For staining, 0.5% crystal violet-20% ethanol solution was overlaid on the washed cells for 15 min at room temperature. The staining solution was removed and the stained cells were washed with water. After drying, plaque numbers were counted to calculate plaque forming unit (PFU) per mL of each rRSVs as viral titer.

### 2.3. Infection and Neutralization of rRSV in 96-Well Cell-Culture Plates

2%FBS/EMEM-1%PS was used as medium for dilution. 96-well cell-culture plates were used for RSV neutralization assay. RSV dilutional controls consisted of 3-fold serial-dilution from 60,000 PFU/well to 9 PFU/well and medium sample with no RSV. RSV neutralization antibody (Ab) manufactured by Denka-Seiken (#300553, Tokyo, Japan) and anti-RSV immune globulin provided by BEI Resource (NR-21973, Manassas, VA, USA) were used as neutralizing antibody serum. Diluted Ab sample consisted of 4-fold serial-dilution from 100-fold dilution to 1638400-fold dilution. After serial dilution, diluted RSV was added to Ab dilutional sample, and the mixture was incubated at 37 °C in 5% CO_2_ for 1 h to neutralize RSV with Ab. Diluted RSV samples were supplied with 4 (24 or 48 h) or 2 (72 h) × 10^4^ cells/well cell suspension collected before experiment, and incubated at 37 °C in 5% CO_2_ for 24, 48 or 72 h. Ab-neutralized samples were added to 2 × 10^4^ cells/well cell suspension collected before experiment, and incubated at 37 °C in 5% CO_2_ for 72 h. Viral titer scores of each well were measured as stated below in the Section 2.4, Section 2.5 and Section 2.6. Each value was calculated as the mean of three independent experiments, each performed in triplicate, in order to assess both intra- and inter-experimental variability. IC50 of Ab sample was calculated with 4PL curve fitting using these measured scores and AAT Bioquest IC50 Calculator (https://www.aatbio.com/tools/ic50-calculator) accessed on 31 January 2025.

### 2.4. Detection of rRSV-Fluorescent Protein Infection and Neutralization

After 1–3 days incubation, cell-supernatants were removed and infected cells were fixed with 4% paraformaldehyde/phosphate buffer solution (PFA/PBS, #09154-85, Nacalai, Kyoto, Japan) for 10 min at room temperature. Fixed cells were washed with D-PBS(-) twice and supplied with D-PBS(-). GFP green fluorescence or mCherry red fluorescence of each well was detected with Envision 2104 Multilabel Reader manufactured by PerkinElmer (Waltham, MA, USA).

### 2.5. Detection of rRSV-Luciferase Neutralization

After 72 h incubation, infected cells were washed with D-PBS(-) and lysed with Lysis buffer (25 mM Tris-HCl (pH 7.8), 8 mM MgCl_2_, 15% glycerol, 1% TritonX-100, 1 mM dithiothreitol (DTT)) for 10 min at room temperature. Lysed cells were mixed with 2 mM D-luciferin Na-1 mM ATP/Lysis buffer, and luminescence of each well was measured with ARVO MX 1420 Multilabel Counter manufactured by PerkinElmer.

### 2.6. Detection of RSV Neutralization Using ELISA

ELISA was performed referring to [14]. After 72 h incubation, cell-supernatants were removed and infected cells were fixed with 80% aceton/20% D-PBS(-) for 15 min at 4 °C. Fixed cells were washed with TBST twice and treated with 10,000-fold dilution of biotinylated α-RSV-F 1st antibody (#MAB8262B-5, Merck, Darmstadt, Germany) for 60 min at 37 °C. After washing with TBST 4 times, the cells were treated with 20,000-fold dilution of streptavidin-HRP 2nd antibody (#SA00001-0, Proteintech, Rosemont, IL, USA) for 60 min at room temperature. After washing with TBST 4 times, SureBlue HRP-substrate solution (#5120-0075, Seracare, Milford, MA, USA) was added in the wells for 10 min at room temperature, and Stop solution (Seracare#5150-0020) was added to stop the reaction, causing the color of the solution to change from blue to yellow. 450 nm-absorbance of each well was measured with Envision 2104 Multilabel Reader manufactured by PerkinElmer.

## 3. Results

### 3.1. Conditions of RSV-Fluorescent Protein Infection

**Figure 2:**
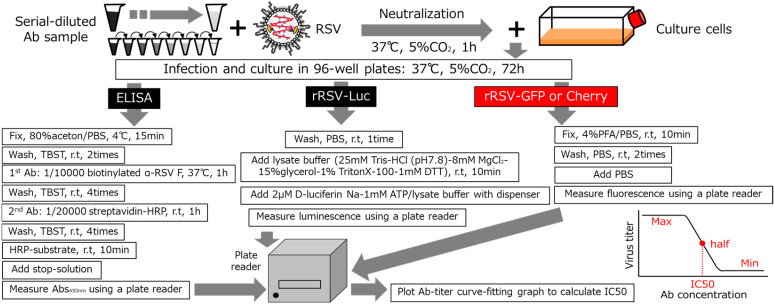
The schematic of RSV neutralization assay protocols.

Although rRSV-Luciferase shows high luminescence signal intensity for RSV growth, its detection requires expensive luciferin reagents and equipment, careful control of temperature and substrate injection. In contrast, rRSV-fluorescent protein, GFP or Cherry, does not require antibody or substrate reagents and machinery injection equipment, but needs fixation and washing of infected cells only (Figure 2). Thus, to construct a high-throughput RSV neutralization assay protocol, we focused on rRSV expressing fluorescent proteins as reporters.

First, we examined conditions of rRSV-fluorescent protein detection during RSV reproduction in Hep-2 cells depending on the multiplicity of infection and other conditions. We focused on the color of 96-well cell culture plates. We chose black color plates and white color plates since black plates have low background fluorescence, while white plates can gain high intensity of fluorescence and luminescence. rRSV-GFP and Cherry were serially diluted with medium in 96-well plates from 60,000 PFU/well to 9 PFU/well using 8-times 3-fold dilution. Medium samples without RSV were used as negative control. RSV dilutional controls were supplied with Hep-2 cells and incubated at 37 °C in 5% CO_2_ for 24, 48 or 72 h. After fixation and washing, fluorescence of each well was detected using plate reader. Measured fluorescence values were normalized by setting the signal from the negative control to 1.0 relative fluorescence unit (RFU), allowing comparison across experimental conditions.

**Figure 3:**
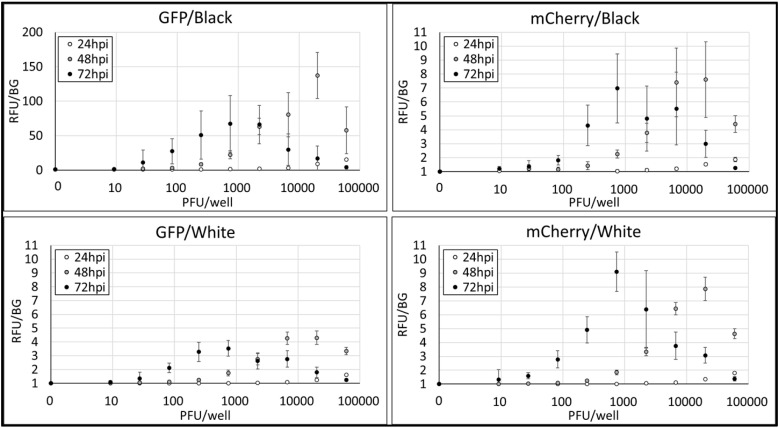
The examination of conditions of rRSV-fluorescent protein infection experiments. X-axis shows rRSV PFU per well. Y-axis shows relative fluorescence unit normalized as 0 PFU sample = 1.0 RFU. Each RFU was calculated as average of three experiments of triplicates per one experiment. Error bars show standard deviations of each samples normalized as 0 PFU sample = 1.0 RFU. The examined parameters were two rRSV reporter types (GFP or Cherry), three incubation times (24, 48, or 72 h post-infection), and two plate types (black or white 96-well plates), resulting in 12 experimental conditions assessed.

Figure 3 shows the results. All conditions showed PFU-dependent fluorescence. 24 h post infection (hpi) showed insufficient peak fluorescence at 60,000 PFU, and 48 or 72 hpi showed sufficient peak fluorescence at 20,000 or 741 PFU. PFU over peaks showed the drop in fluorescence, suggesting the effect of cell death by high MOI. Following the common view that black plates have lower background fluorescence than white plates, in the condition of rRSV-GFP, white plates showed lower 72 hpi peak RFU (3.54) than those black plates showed (67.1). rRSV-Cherry showed similar 72 hpi peak RFU in both colors of plates (black: 6.98, white: 9.10). Focusing on the consistency of RFU, black plates tended to have higher standard deviations showed by error bars than white plates have, which means that black plates show unstable fluorescent measurement compared to white plates. Considering fluorescent intensity, consistency and rRSV stocks consumption, we chose 72 hpi, 200 PFU per well and white plates for the condition of next RSV neutralization assay experiments.

### 3.2. Comparison of RSV Neutralization Assays Using ELISA or rRSV-Reporter Proteins

Next, we compared RSV neutralization assay protocols using ELISA or rRSV-reporter proteins. In 96-well plates, manufactured RSV neutralization Ab was diluted 4 times serially from 100-fold dilution to 1,638,400-fold dilution. 200 PFU per well rRSV-dilution was added to Ab dilutions, and they are incubated 1 h for neutralization. Hep-2 cells were seeded into samples. After 72 h culture, samples were measured as described above. Briefly, rRSV-WT ELISA samples were fixed, treated with 1st and 2nd antibody 1 h per an antibody, detected with yellow coloration of substrate solution of HRP conjugated with 2nd antibody. rRSV-GFP or Cherry samples were fixed, washed and fluorescence of GFP or mCherry was quantified. rRSV-Luc samples were lysed and luminescence was quantified by addition of luciferin, substrate of luciferase.

**Figure 4:**
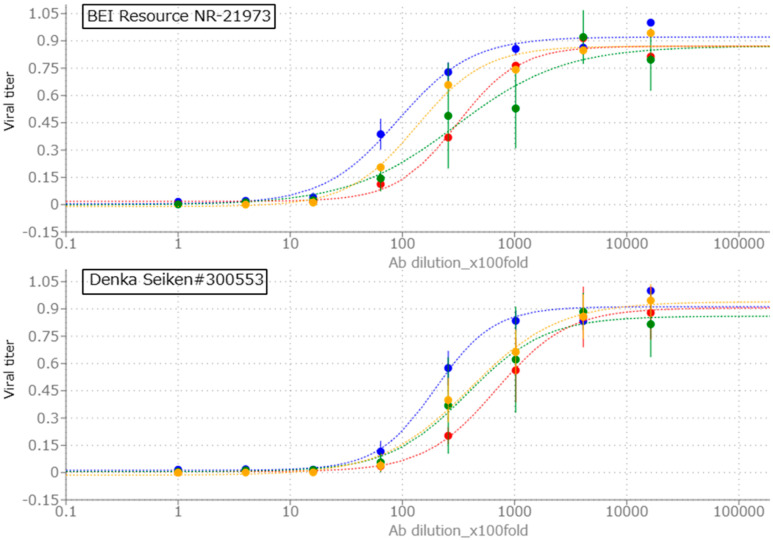
The examination of conditions of rRSV neutralization assay. X-axis shows dilutional fold of α-RSV antibody (Ab). Y-axis shows relative fluorescence unit (RFU; GFP and Cherry), relative luminescence unit (RLU; Luc) or relative absorbance of 450 nm wavelength (Abs450 nm; ELISA) normalized as maximum viral score = 1.0. Each score was calculated as average of three experiments of triplicates per one experiment. Error bars show standard deviations of normalized each score. Total 8 conditions were examined; RSV antibody control samples (BEI resource NR-21973 and Denka Seiken #300553) and rRSV (blue: WT for ELISA, red: Cherry, green: GFP and yellow: Luc).

Figure 4 shows the results. All tested assay conditions demonstrated antibody dependent RSV neutralization, reflected by a reduction in the viral titer signal (RFU for GFP and Cherry, RLU for luciferase, and Abs450 nm for ELISA). For comparison across conditions, all values were background-subtracted and normalized to a maximum signal of 1.0. rRSV-WT ELISA and rRSV-Cherry showed higher consistency of viral titer scores compared to rRSV-Luc and rRSV-GFP conditions. For each assay condition, IC50 values were determined as the dilution of antibody reducing the background-corrected viral titer signal to 50% of its normalized maximum. BEI resource NR-21973 showed log2IC50 scores (ELISA: 13.15, GFP: 14.88, Cherry: 14.91, Luc: 13.71), and Denka Seiken #300553 showed log2IC50 scores (ELISA: 14.27, GFP: 15.20, Cherry: 16.06, Luc: 15.29), suggesting that rRSV-WT ELISA neutralization assay has similar ability of Ab-titration to ELISA assay used in previous research [14]. Although each rRSV showed different IC50 score, rRSV-Cherry and ELISA showed similar relative log2IC50 scores focusing on the score ratio of #300553 per NR-21973 (ELISA: 2.17, GFP:1.24, Luc: 2.99, Cherry: 2.22), suggesting that like RSV ELISA neutralization assay, rRSV-Cherry neutralization assay can convert IC50 score of clinical samples for comparison using RSV neutralizing antibody control samples.

## 4. Discussion

This paper shows the examination of rRSV neutralization assay protocol different conditions, such as plates, cells and reporter genes. Information on direct comparison of such conditions in fluorescent-based neutralization assays is currently limited. Therefore, in this study, we compared and discussed the current available neutralization assays based on different technical conditions and practical aspect with our new established assay. Although black plates are usually used for fluorescent assay because of lower background fluorescence than white plates, this study showed that white plates showed high enough and stable fluorescence of rRSV-fluorescent proteins, which enable to be applied for fluorescent assay instead of black plates. White plates also have high visibility of medium surface color in each well, which is useful to prevent mistakes of sample preparation when a lot of plates are prepared for epidemiological surveillance with manual operation. Both rRSV-GFP and rRSV-Cherry produced detectable fluorescence proportional to viral growth. Neutralization by control RSV antibody was observed across all rRSV-based assays as a reduction of the corresponding reporter signal, measured as fluorescence (GFP or Cherry), luminescence (Luciferase), or absorbance (ELISA). Especially, rRSV-Cherry assay showed high possibility to measure neutralizing titers of clinical samples for surveillance like ELISA assay, which should be examined in future studies using real clinical samples.

Considering simultaneous measurement of many plates at once for surveillance assay, saving time and cost is a very important perspective. Although ELISA protocol had high stability with small standard deviations, ELISA needs a long time for two antibody-treatments and preparation of reagents like 1st antibody, 2nd antibody and substrate solution of enzyme conjugated with 2nd antibody, which can be skipped by rRSV fluorescent protocol. Though rRSV-Luc had high signal intensity with very low background, it also had high instability of luminescence with large standard deviations. This point may be improved using smaller luciferase like previous study [19]. Furthermore, rRSV-Luc detection needs 96-times dispensing of substrate solution, shaking solution and interval time to complete mixing solution in each well just before measurement of luminescence to measure one 96-well plate. Even when these steps take 10 s per well, luminescence measurement takes 16 min per one 96-well plate with usual single-well injector and detector. This detection takes longer time especially when numerous plates are examined in one assay. To solve this problem, specialized luminescent plate readers equipped with multi-well injectors and detectors are necessary. In contrast, measurement of fluorescence in a 96-well plate only takes ~1 min per plate, shorter time than luciferase luminescence, which is one of advantages of rRSV-fluorescent protein neutralization assay for multiple plates assay. Red fluorescence of mCherry is said to be useful to avoid cellular autofluorescence rather than green fluorescence of GFP overlapped to wavelength of NAD(P)H [20], so rRSV-Cherry is appropriate for a tool to measure RSV titer using RSV-infected cells. Different from conventional assays using plaque counting, qPCR or ELISA, rRSV-Cherry neutralization assay does not need complicate and time-consumable preparation of reagents including antibody or substrate solution (Table 1), which may be helpful to standardize RSV neutralization assay protocol varied in each laboratory [21,22].

Although the current assay is proposed as a low-cost and accessible alternative, it still requires handling of live RSV and therefore a minimum of BSL-2 containment. This implicates specialized infrastructure, approvals, and trained personnel, conditions which may not easily be met in some diagnostic or low-resource settings. Due to the nature of the experiments, we were not able to show statistically significant values in selecting the condition of the assay. In addition, we did not include testing the actual samples from surveillance or clinical studies. Future studies demonstrating the practical usage of the study assay will be important.

In Fluorescent Assays, it is well known that Black plates absorb light, which minimizes background and crosstalk in fluorescence measurements. On another hand, White plates reflect light, which can increase background and crosstalk in fluorescence measurements. The fluorescent signal will also depend on the fluorescent protein and the PFU/well used in the assay. According to previous research, recombinant bovine respiratory syncytial virus, which had mCherry gene between viral P and M genes, showed diffuse fluorescence in the infected cells, compared to fluorescent labelling of viral F protein [23], suggesting that rRSV-Cherry might be more affected by localization of expressed mCherry in the cells compared to another protocols using antibody or substrate reagents. This may be the reason why black or white plates and GFP or mCherry proteins showed different variabilities, and a key to improve rRSV-Cherry neutralization assay protocol for more stable measurement. Some laboratories generated fluorescent protein-expressing recombinant RSV derived from not only laboratory strains but also clinical strains [24,25]. Further development of rRSV-Cherry may improve its usability for neutralization assay, such as stability of fluorescence, antigenicity to clinical strains for clinical studies or vaccine development, and dispensation system of rRSV-fluorescent protein for clinical institutes worldwide.

## 5. Conclusions

In conclusion, this paper shows the improved RSV neutralization assay protocol using rRSV-Cherry, 3 days culture and white 96-well plates. This protocol is time and cost-saving, and is suitable for RSV epidemiological surveillance analyzing a large set of samples without specialized or numerous expensive machines for detection.

## Figures and Tables

**Table 1:** Comparison of RSV neutralization assay protocols in previous or our studies.

Protocol	Plaque Counting [8,9,10]	qPCR [11]	ELISA [12]	rRSV-Cherry in This Study
RSV	EGFP-expressing rRSV [8] or RSV A2 strain [9,10]	RSV A2 or RSV B1	RSV A2 or long or B (18537)	rRSV-Cherry
Cell line	Vero [8,10] or A549 [9]	Vero	Hep2 or Vero or A549	Hep2
Detection	Plaque counting with ELISPOT reader [8,9,10] + Immunostaining [9,10]	SYBR-green dye fluorescence	Absorbance 450 nm of HRP-substrate solution	Reporter fluorescence
Reagents	Wash buffer, fixation buffer and antibody reagents	RNA extraction kits, SYBR-green qPCR reagents and primers for RSV N gene	Wash buffer, fixation buffer, antibody reagents and HRP-substrate solution	Wash buffer and fixation buffer
Instruments	Plate reader specialized for ELISPOT assay	qPCR thermal cycler	Plate reader for absorbance	Plate reader for fluorescence

## Data Availability

The data used to support the findings of this study are available upon request from the corresponding author.
